# A physiotherapy-led transition to home intervention for older adults following emergency department discharge: protocol for a pilot feasibility randomised controlled trial

**DOI:** 10.1186/s40814-021-00954-5

**Published:** 2022-01-03

**Authors:** Mairéad Conneely, Aoife Leahy, Margaret O’Connor, Louise Barry, Gillian Corey, Anne Griffin, Íde O’Shaughnessy, Ida O’Carroll, Siobhán Leahy, Dominic Trépel, Damian Ryan, Katie Robinson, Rose Galvin

**Affiliations:** 1 School of Allied Health, Faculty of Education and Health Sciences, Ageing Research Centre, Health Research Institute, University of Limerick, Limerick, Ireland; 2 Department of Ageing and Therapeutics, University Hospital Limerick, Dooradoyle, Limerick, Ireland; 3 School of Nursing and Midwifery, Faculty of Education and Health Sciences, University of Limerick, Limerick, Ireland; 4 Department of Sport, Exercise & Nutrition, School of Science & Computing, Galway-Mayo Institute of Technology, Dublin Road, Galway, Ireland; 5 Trinity Institute of Neurosciences, School of Medicine, Trinity College Dublin, Dublin, Ireland; 6 Limerick EM Education Research Training (ALERT), Emergency Department, University Hospital Limerick, Dooradoyle, Limerick, Ireland

**Keywords:** Older adults, Emergency department, Adverse outcomes, Integrated care, Feasibility randomised controlled trial

## Abstract

**Background:**

Older adults frequently attend the emergency department (ED) and experience high rates of adverse outcomes following ED presentation including functional decline, ED re-presentation and unplanned hospital admission. The development of effective interventions to prevent such outcomes is a key priority for research and service provision. This paper reports a protocol designed to evaluate the feasibility of conducting a three arm randomised controlled trial (RCT) within the ED setting and in the patient’s home. The interventions are comprehensive geriatric assessment (CGA), ED PLUS and usual care.

**Methods:**

The ED PLUS pilot trial is designed as a feasibility RCT conducted in the ED and Acute Medical Assessment Unit of a university teaching hospital in the mid-west region of Ireland. We aim to recruit 30 patients, aged 65 years and over presenting to the ED with undifferentiated medical complaints and discharged within 72 h of index visit.

Patients will be randomised by a computer in a ratio of 1:1:1 to deliver usual care, CGA or ED PLUS during a 6-month study period. A randomised algorithm is used to perform randomization. CGA will include a medical assessment, medication review, nursing assessment, falls assessment, assessment of mobility and stairs, transfers, personal care, activities of daily living (ADLs), social supports and baseline cognition. ED PLUS, a physiotherapist led, multidisciplinary intervention, aims to bridge the transition of care between the index visit to the ED and the community by initiating a CGA intervention in the ED and implementing a 6-week follow-up self-management programme in the patient’s own home following discharge from the ED. The outcomes will be parameters of the feasibility of the intervention and trial methods and will be assessed quantitatively and qualitatively.

**Discussion:**

Rising ED visits and an ageing population with chronic health issues render ED interventions to reduce adverse outcomes in older adults a research priority. This feasibility RCT will generate data and experience to inform the conduct and delivery of a definite RCT.

**Trial registration:**

The trial was registered in Clinical Trials Protocols and Results System as of 21^st^ July 2021, with registration number NCT049836020.

**Supplementary Information:**

The online version contains supplementary material available at 10.1186/s40814-021-00954-5.

## Background

Population ageing poses challenges to the healthcare system with older adults requiring acute health care services at an increasing rate [1], particularly for emergency department (ED) services [2–5]. EDs are complex environments in which to provide care to older adults [6, 7]. Older adults are more likely to experience longer ED lengths of stay, have more complex presentations and higher rates of adverse outcomes following discharge from the ED than younger people [8–10]. Adverse outcomes include functional decline, poorer quality of life, unscheduled return visits to the ED, hospitalisation and mortality [11–13]. Given the risk of adverse outcomes following an index visit, the development of effective interventions is a key priority for research and service provision.

A presentation to an ED can be viewed as an opportunity to assess those at risk of adverse outcomes and initiate a care plan in those deemed as ‘high risk’ [2, 14]. Consequently, a number of interventions have been examined to reduce the incidence of adverse outcomes among ‘high risk’ older adults following presentation to the ED [15–18]. The evidence from systematic reviews regarding the effectiveness of these interventions is mixed given the heterogeneity in study designs, description of the interventions and outcomes assessed. In line with Medical Research Council (MRC) Guidelines [19, 20], we developed an evidence base by completing an umbrella review of the effectiveness of ED initiated interventions to improve outcomes in older adults. This comprehensive umbrella review included nine systematic reviews representing 29 randomised controlled trials (RCTs) investigating various interventions including implementation of care pathways based on risk profiling, nurse-led interventions, case management within the ED and post-discharge, comprehensive geriatric assessment (CGA) and discharge planning [17]. These interventions were either ED-based interventions or ED-initiated interventions with community follow up, termed ED-community transitional services interventions [16, 17]. This umbrella review identified low-quality evidence to support ED interventions in reducing functional decline, improving patient experience and improving quality of life. The quality of evidence of the effectiveness of ED interventions to reduce mortality and ED revisits varied from very low to moderate. There was no effect of any of the interventions on hospital admissions after the ED index visit. No systematic review reported the outcome of length of ED stay. The authors of all systematic reviews included in this umbrella review recommend that more high quality RCTs need to be conducted in this area.

One of the most commonly investigated interventions in older adults is CGA, which is considered the gold standard approach to improving a range of outcomes for frail older adults in acute hospitals [21]. There is evidence that a treatment plan based on CGA can increase the chance of living independently at home, and have a positive effect on physical function outcomes compared to usual care in various other care settings, including hospitalised older adults [21–23], but the evidence base for its implementation in the ED is insufficient [24, 25]. Thus, clinicians have limited evidence on which to base their practice. There is a need for further pragmatic trials to assess the effectiveness of CGA in an ED setting.

Our umbrella review did not identify any systematic review that explored an ED intervention targeting older adults led by health and social care professionals (HSCPs) such as physiotherapists, medical social workers and occupational therapists. Research has demonstrated that HSCPs can play a role in the ED in improving patient experience, reducing length of ED stay and preventing hospital admissions in other age groups [26–28].

HSCPs are well placed to deliver interventions to reduce functional decline among older adults. Furthermore, physiotherapists are key HSCPs who lead interventions to improve functional decline and address other adverse outcomes in hospital and community settings [13, 29–31]. Integrated care programmes which include screening to identify needs, linking to community services and monitoring with a primary care provider, have been advocated to improve the continuum of care from the ED into the community [32–35].

A key research priority defined by Kings Fund and Veteran Affairs Reports is to develop user informed transitional interventions to bridge the ED and community [16]. We developed a novel intervention in response to the findings from our umbrella review to address service needs and key geriatric research priorities outlined in the literature [36–39]. The proposed intervention, ED PLUS, is a user-informed, self-management programme developed based on best available evidence to improve functional decline in older adults. ED PLUS, a physiotherapist-led, multidisciplinary intervention, bridges the transition of care between the index visit to the ED and the community by initiating a CGA intervention in the ED and implementing a 6-week follow-up self-management programme in the patient’s own home following discharge from the ED. This feasibility RCT will generate data and experience to inform the conduct and delivery of a definite RCT.

### Aims and objectives

The overall aim of this work is to examine the feasibility of implementing a 6-week transitional intervention delivered both face to face and via telephone support for older adults discharged from the ED.

Primary aims are:

1. To establish whether the intervention is acceptable to older adults discharged from the ED
2. To assess the feasibility of undertaking a definitive RCT of the ED PLUS intervention in this population, evaluating the process of recruitment, screening, randomisation, and collection of data at baseline and outcome data
3. To explore any trial design aspects that may require refinement prior to proceeding to a full RCT

Secondary aims are:

1. To examine the effect of ED PLUS on functional decline at 6 weeks and 6 months
2. To explore the effect of the ED PLUS on quality of life at 6 weeks and 6 months
3. To examine the effects of ED PLUS on process outcomes (ED revisits, unplanned admission, length of stay) at 6 weeks and 6 months

## Methods

### Trial design

This is a parallel group pilot RCT with a 1:1:1 allocation ratio. In order to ensure standardised conduct and reporting, the Standard Protocol Items for Intervention Trials (SPIRIT) guidelines will be used [40]. Table 1 describes the feasibility/piloting phase of the MRC Framework alongside the activities involved in this process evaluation [41]. The trial is registered at Clinical trials.gov (NCT04983602). Ethics approval was obtained from the HSE Mid-Western Area Research Ethics Committee (088/2020).

**Table 1:** Mapping activities to Medical Research Council framework section 2 assessing feasibility and pilot methods

2	Assessing feasibility and piloting methods	
2.1	Testing procedures for acceptability, compliance, and intervention delivery	Tested components for feasibility and acceptability. Acceptability of the ED PLUS intervention discussed with PPI panel. Assess feasibility of delivering intervention via face-to-face intervention and via telephone in terms of recruitment, retention and usability through a pilot of 6 weeks with 10 participants. Assess acceptability through qualitative interviews.
2.2	Estimating recruitment and retention	Recruitment from an ED and AMAU of a large, single tertiary care facility. Consult with PPI group of older adults to determine best practice for ongoing retention of trial participants. Consult with trial methodology groups (e.g. Health Research Board Trial Methodology Research Network) and working groups to determine the best methodology for ongoing retention of trial participants.
2.3	Determining sample size	The results will be used to inform the sample size of a future definite RCT.

*AMAU* Acute medical assessment unit, *ED* emergency department, *PPI* public and patient involvement

### Trial setting

The pilot RCT will be conducted in the ED and Acute Medical Assessment Unit (AMAU) of a university teaching hospital in the mid-west region of Ireland. University Hospital Limerick (UHL) serves both rural and urban areas of Limerick, Clare and North Tipperary. This is a university teaching hospital, which caters for the general medical, surgical and emergency treatment of patients in its catchment area of 470,000 people. UHL has a 24 h, seven days a week ED that functions 365 days a year and is a tertiary referral centre for the mid-west region.

### Participants

#### Sample size

As this is a pilot feasibility RCT, the primary aim is not to evaluate the clinical effectiveness; therefore, we will not undertake a formal power analysis for sample size for a primary outcome [42, 43]. We aim to recruit 30 participants over a two-month period as recommended for pilot studies [44, 45]. From other studies in similar settings, we anticipate a drop-out rate of 10% in total for the three groups [46, 47]. Data obtained from this pilot RCT will inform the power analysis for a definitive trial.

#### Eligibility criteria

The inclusion criteria for participants are adults aged over 65 years of age with undifferentiated medical complaints presenting to an ED. Participants must:

- Be medically stable as deemed by the treating physician (vital signs are within normal limits, patients do not require a surgical assessment) [48]
- Have a score of ≥ 2 on the Identification of Seniors at Risk (ISAR) screening tool [49, 50]. The ISAR is a validated screening tool for use in the ED to detect older adults at risk of adverse outcomes including functional decline, revisits to the ED, unplanned hospitalisation and mortality within 6 months of the ED presentation. A cut-off of ≥ 2 is recommended [49].
- Be community dwelling
- Have a short-term ED admission with a length of stay of ≤ 72 h from ED presentation.

Exclusion criteria include:

- Individuals under the age of 65 years
- Have a score of <l 2 on the ISAR
- Older adults who present with acute myocardial infarction, stroke or non-medical problems or require a surgical assessment or psychiatric issues including schizophrenia
- Older adults who are medically unstable
- If neither the patient nor carer can communicate in English sufficiently to complete consent or baseline assessment
- Older adults who are admitted to hospital from the ED Older adults who have a confirmed positive COVID-19 test on presentation to the ED

### Recruitment

Patients will be screened for inclusion between 8 am and 4 pm Monday to Friday. Older adults who score ≥ 2 on the ISAR will be asked if they would like to participate in the trial by a dedicated research nurse during their ED or AMAU visit. They will be given an opportunity to read the information leaflet and ask any questions they may have before giving written informed consent if agreeable. Potential participants will be approached regarding trial recruitment post ED triage thereby ensuring rapid assessment shortly after hospital arrival by the dedicated SOLAR team in the ED. The SOLAR team (consisting of a consultant in geriatric medicine/geriatric specialist registrar, specialist geriatric nurse, senior pharmacist, senior physiotherapist, senior occupational therapist and senior medical social worker) in the ED will assess all participants in the intervention group and perform CGA.

**Fig. 1:**
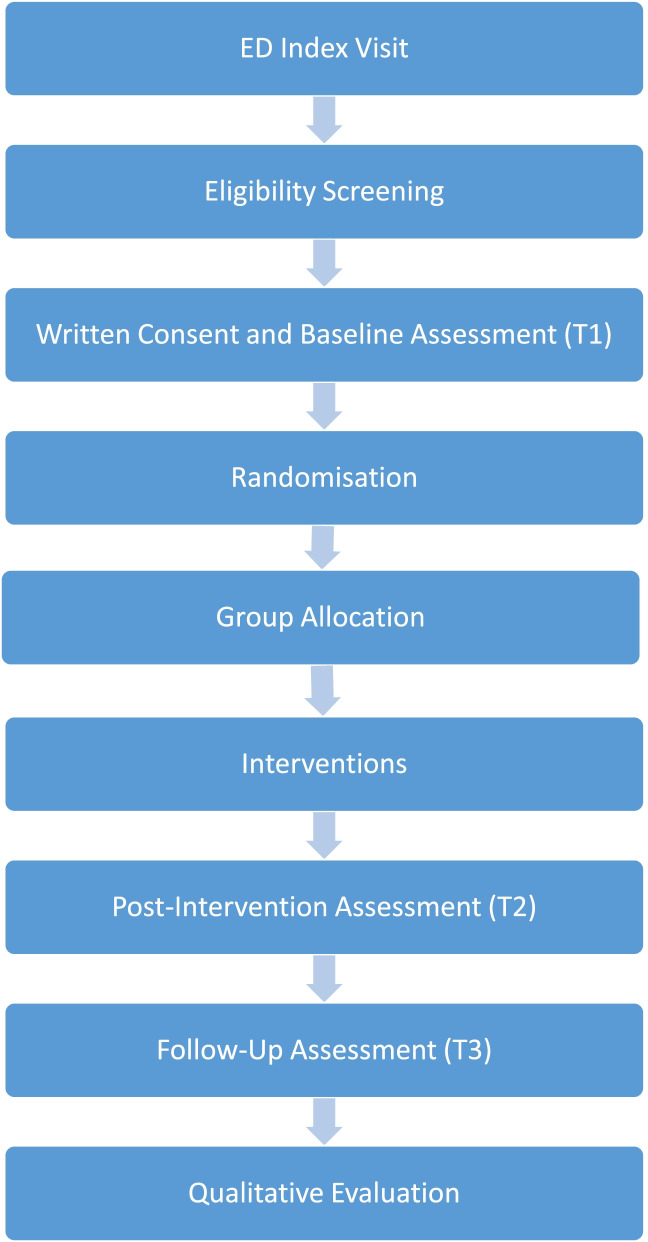
Study design

Figure 1 outlines the trial design.

### Randomisation and allocation concealment

Eligible participants will be block randomised in groups of 30 participants using a computer generated 1:1:1 allocation through a Sealed Envelope website (www.sealedenvelope.com). Allocation will be concealed from participants and researchers until after consent has been obtained and baseline evaluations performed. The research nurse (GC) will assign participants to interventions once allocation has been revealed.

### Blinding

Due to the nature of the intervention, trial participants and treating clinicians are unable to be blinded to group allocation. The outcome assessor will be blinded to group allocation. The outcome assessor has no access to the medical files of the participants and will be given a list of questions related to the outcome measures to ask each participant at 6 weeks and 6 months.

### Informed consent

Potential participants will initially be informed of the study by the triage nurse or the treating physician. If participants indicate a willingness to hear more about the trial, the dedicated research nurse (GC) on the project will be informed. The research nurse will subsequently meet with the potential participant, describe the study in detail and provide potential participants with an information leaflet. Participants will be given time to consider the trial and to ask questions. Written informed consent will be obtained by the research nurse if/when the participant indicates their willingness to formally participate in the study. Participants will have the duration of their index admission to consider participation in the trial. Participants can withdraw at any stage without any negative implications for their treatment.

### Interventions

There are three arms to this pilot feasibility trial: usual care, intervention arm 1 (CGA) and intervention arm 2 (ED PLUS).

#### Usual Care

The comparison group will receive routine care as would be usual in the ED. At present, there is no dedicated team to perform CGA in the ED and AMAU at UHL, but an interdisciplinary HSCP team is available at the discretion of the referring ED doctor or medical team. This process will be continued during the study and will be documented.

#### Intervention arm 1 comprehensive geriatric assessment

The intervention arm 1 will comprise initially of a detailed interdisciplinary assessment and intervention by one or more members of the dedicated SOLAR team in UHL.

Comprehensive geriatric assessment (CGA) will include but not be limited to a medical assessment, medication review, nursing assessment, falls assessment, assessment of mobility and stairs, transfers, personal care, activities of daily living (ADLs), social supports and baseline cognition. Members of the SOLAR team will be guided by their clinical expertise and codes of professional practice. Similarly, interventions prescribed by the SOLAR team will be based on subjective and objective assessment of patients and will include medication alterations, lifestyle advice, prescription of mobility aids, provision of home exercise programmes and onward community referral as appropriate. All assessments and interventions will be included in the medical chart of individual participants. All healthcare professionals will adhere to guidelines for COVID-19 throughout the duration of the intervention. Table 2 outlines the core components of CGA.

**Table 2:** Components of Comprehensive Geriatric Assessment

Medical	Co-morbid conditions and disease severity Medication review Nutritional status Problem list
Mental health	Cognition Mood and anxiety Fears
Functional capacity	Basic activities of daily living Gait and balance Activity/exercise status Instrumental activities of daily living
Social circumstances	Informal support from family or friends Social network such as visitors or daytime activities Eligibility for being offered care resources
Environment	Home comfort, facilities and safety Use or potential use of tele-health technology, etc. Transport facilities Accessibility to local resources

#### Intervention arm 2 ED PLUS

The ED PLUS programme consists of CGA in the ED plus a 6-week multifactorial, multidisciplinary, patient centred self-management support and exercise programme. The team consists of a geriatrician, dietitian, occupational therapist and led by a physiotherapist. It is structured to maximise the patient’s self-efficacy and support independence in functional activities and provide a continuum of care from the ED to the patient’s home. ED PLUS aims to address issues with mobility, strength, balance, malnutrition, medication adherence, fatigue and enable self-management. The development of ED PLUS has been informed by the best available theoretical evidence on the conduct of feasibility trials of complex interventions [51]. A synthesis of the literature was undertaken and input was also provided from geriatricians, geriatric nurses, dietitians, occupational therapists, physiotherapists and a PPI panel of older adults. Implementing care to older adults is complex due to the presence of multimorbidity [37]. This programme is designed with the latest evidence base on the management of people with multiple chronic conditions [52], in particular a 6-week occupational therapy-led, multidisciplinary, self-management programme for community dwelling people with multimorbidity [53]. Furthermore, the existing evidence base supports the appointment of one clearly defined health care professional who co-ordinates care, a focus on patient preferences, shared decision making and a focus on functional outcomes [52]. An intervention co-ordinated by a defined HSCP has shown to be to be successful in improving clinical outcomes among frequent ED users [54].

**Fig. 2:**
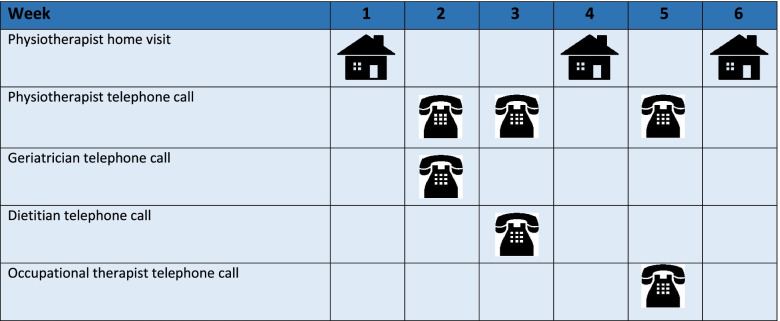
Frequency of ED PLUS sessions

The 6-week programme will involve a telephone call from the discharging member of the SOLAR team in the ED to the lead physiotherapist (MC) who will subsequently contact the patient to arrange a physiotherapist home visit. The home visit will take place 24–48 h post ED discharge and involve an initial assessment and the setting of patient goals over the 6-week period. The programme will involve three home visits by a physiotherapist over a period of 6 weeks with telephone support in between the visits by the physiotherapist. The other members of the MDT will telephone the patient as outlined in Fig. 2. The physiotherapist will implement a personalised treatment plan liaising with the geriatrician with key regard to medication adherence, exercise prescription, physical activity and strength. ED PLUS incorporates the most recent evidence on physical activity and falls prevention, duration of physical activity programmes, management of multimorbidity and physical exercise interventions for improving measures of physical function in older adults [31, 52, 55–57]. The evidence for the benefits of exercise in improving age-related decline and preventing falls are significant [56]. Evidence exists that physiotherapists help prevent falls following acute rehabilitation [58] with The New Physical Activity Guidelines for Health in the UK have strength and balance activities recommended for older adults who are frail [59].

As outlined in Fig. 2, the geriatrician will conduct a telephone call with the participants to discuss medication, medication adherence and address concerns regarding same. The dietitian will offer a telephone one-to-one dietetic sessions to optimise dietary intake, ensuring adequate energy, protein and micronutrient status given the relationship of malnutrition and functional status [33, 60, 61]. There is also evidence of a link between malnutrition and poorer quality of life, increased risk of hospital admissions and a greater likelihood of admission [62]. The occupational therapist will discuss with the patient regarding self-management including management of morbidity, fatigue and energy conservation to ensure ongoing engagement in physical activity programmes and leisure pursuits. The physiotherapist will act as the key case worker and support the patient during the 6-week programme to address any issues related to their health with liaison with the patient’s doctor as required. Healthcare professionals will adhere to guidelines for COVID 19 throughout the duration of the intervention. Table 3 outlines the ED PLUS intervention and Fig. 2 illustrates the frequency of the sessions.

**Table 3:** Components of ED PLUS

Administration timeline	Personnel	Description
Week 1	Physiotherapist visiting the patients home	Physiotherapy assessment and action plan including individualized goal setting based on the CGA conducted in the ED and the physiotherapy assessment. The physiotherapist performs an assessment of gait, balance, upper and lower limb strength assessment, assesses the patients ability to function independently. An exercise programme is tailored to the patient (examples attached) https://www.nhsinform.scot/healthy-living/preventing-falls/keeping-well/strength-and-balance-exercises https://theros.org.uk/information-and-support/osteoporosis/living-with-osteoporosis/exercise-and-physical-activity-for-osteoporosis/
Week 2	Medication assessment and medication action plan Via telephone Physiotherapist via telephone call	The geriatrician will assess the medication the patient is taking as well as make recommendations for tapering of medication and cessation. Physiotherapist will advise on progressions of exercise programme as appropriate.
Week 3	Nutritional assessment with a dietitian via telephone. Physiotherapist via telephone call	Nutritional status will be determined using validated screening tools and a nutritional care plan will be implemented. Physiotherapist will advise on progressions of exercise programme as appropriate.
Week 4	Physiotherapist visiting the patients home	The physiotherapist reassess the patient and progresses as appropriate, discuss any concerns with the patient and action plan as appropriate
Week 5	Occupational Therapist via telephone Physiotherapist via telephone call	The occupational therapist (OT) will discuss with the patient regarding self-management based on the individual goals set by the patient and physiotherapist. These strategies may include: • Fatigue and energy management • Managing stress and anxiety and maintaining mental health and well-being Physiotherapist will advise on progressions of exercise programme as appropriate.
Week 6	Physiotherapist will visit patients home to conclude the sessions with a focus on review of patient goals and action plan to follow.	Input from all HSCPs is collated for each patient to conclude their ED PLUS management.

### Patient and public involvement

The programme was developed in conjunction with a local public and patient involvement (PPI) panel of older adults in the mid-west/UHL catchment area; this panel was set up to support researchers in geriatric emergency medicine research [63]. To ensure the relevance of research outcomes, it is essential that the views and experiences of older people are taken into account when designing research [16]. The lead author (MC) conducted telephone conversations with representatives from the panel (*N* = 5) to initiate interest in such a trial, assist in the formation of patient centred outcomes, inform the best mode of delivery and the preparation of a patient information booklet. The PPI panel of older adults advised of conducting the physiotherapy assessment in the patients’ home as soon as possible post discharge. The panel also concurred with evidence on a key HSCP to co-ordinate care and to limit the number of HSCPs visiting the home. In order to foster independence, the panel recommended a combination of face-to-face sessions as well as telephone support for patients.

### Criteria for discontinuing or modifying allocated interventions

CGA and ED PLUS are dynamic processes and may change depending on the patient’s presentation. Participation in the trial is voluntary. Participants will be free to withdraw consent and leave the study at any time without giving a reason and without affecting their care. Should a patient withdraw consent to participate in the study, we will seek clarification on whether withdrawal is from a particular element of the study, for example, participation in the CGA, baseline data assessment or access to healthcare records. Data will be irrevocably deleted from the database, in line with participants’ choice of withdrawal. In cases where participants do not respond to follow-up assessments, outcome data that do not involve participant contact (e.g. data from hospital database) will continue to be collected in these cases.

### Strategies to improve adherence to interventions

A number of strategies have been implemented to support adherence to the intervention. A detailed protocol has been developed and shared with the SOLAR team. Daily briefings/team meetings are planned to troubleshoot queries or concerns by the team. Training on the procedures around participant consent has been conducted. A comprehensive and detailed delegation log has been developed. The ED PLUS programme was developed with the PPI group of older adults regarding the delivery of the intervention and the content thus this is a user-informed intervention as per best practice [16].

#### Relevant concomitant care permitted or prohibited during the trial

Participants will be under the medical care of their treating physician for the duration of their ED stay. All relevant consultant stakeholders will continue to provide clinical governance for the patient as per standard clinical practice.

#### Provisions for post-trial care

A detailed letter describing the ED PLUS programme will be sent to the general practitioner (GP) and other HSCPs as required for all intervention group participants discharged directly from the ED. All other patients will have the usual hospital discharge letter provided on discharge.

### Data collection and outcomes

The following data will be collected during the trial:

1. Feasibility outcomes: data from screening, recruitment and follow-up logs will be used to generate realistic estimates of eligibility, recruitment, consent and follow-up rates.

Feasibility will be described in term of recruitment rates, adherence, retention and acceptability of ED PLUS. Recruitment rates will be described at the percentage of eligible study population who consent to participate in ED PLUS. Adherence will be recorded as the number of home visits made by the physiotherapist in the ED PLUS team, plus the number of interactions with the geriatrician, dietician, physiotherapist and occupational therapist.

Retention will be defined as the percentage of enrolled participants completing the post-intervention assessment at 6 weeks. Acceptability of the intervention will be determined through the qualitative evaluation interviews. For the ED PLUS, face to face visits will be recorded and adherence will be calculated as a percentage of the total number of visits. These data alongside the qualitative interviews will be used to measure adherence and to assess whether any modifications are required to improve engagement.

2. Clinical outcome measures
  1. Functional status using the Barthel Index [64]. The Barthel Index is the most commonly used tool for assessment of functional outcome in older adults in clinical settings [65, 66]
  2. Quality of life using the EuroQoL-5D EQ-5D-5L [67], a standardised measure of health status
  3. ED revisit assessed via medical records and patient recall and nursing home admission within 6 weeks and 6 months of initial index visit
  4. Unplanned hospitalisation nursing home admission assessed via patient recall and medical records within 6 weeks and 6 months of initial index visit
  5. Mortality
  6. Healthcare utilisation will also be captured at 6 weeks by telephone contact

**Fig. 3:**
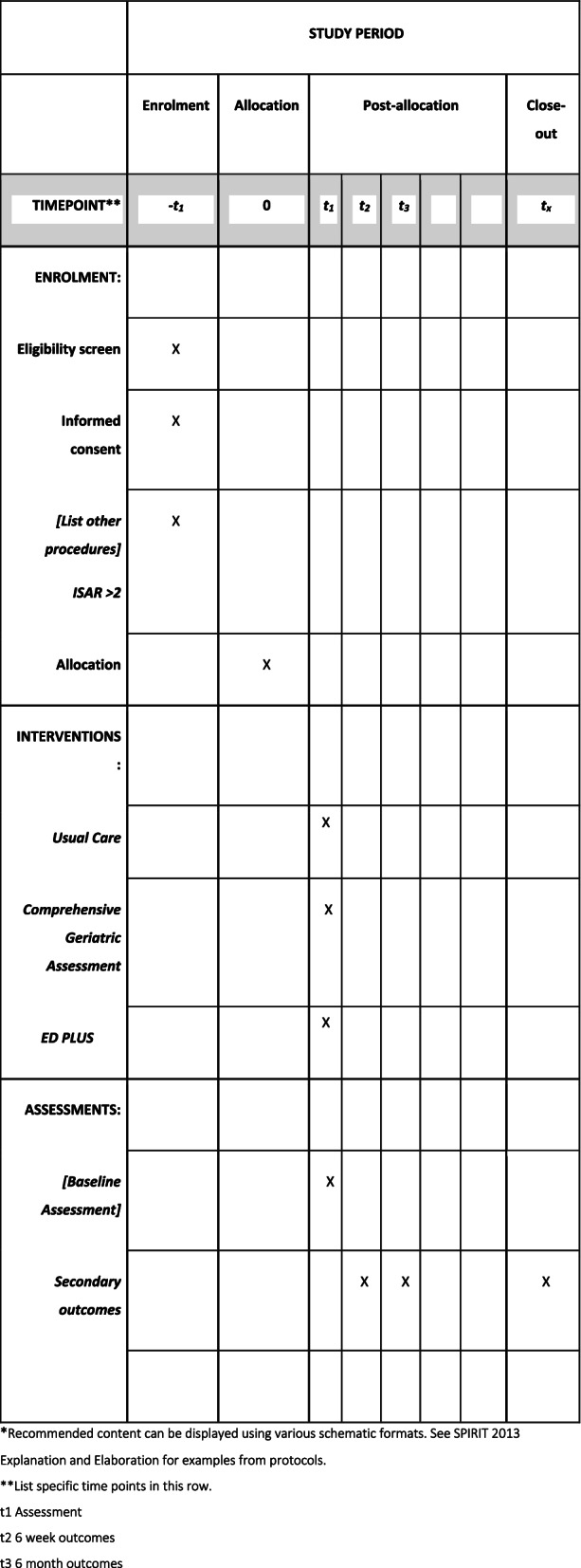
Example template of recommended content for the schedule of enrolment, interventions and assessments*

Follow-up at 6 weeks and 6 months will be captured by telephone contact by a research nurse who is blinded to group allocation (Fig. 3).

### Economic evaluation

Given the stage of development of ED PLUS, economic evaluation will primarily seek to examine and help establish resources required for this complex intervention. This early-stage economic evaluation will work alongside the pilot feasibility RCT and will measure resource intensity, will help establish important intangibles (e.g. non face-to-face time related to intervention delivery) and will determine correct ways to assign salient costs to resources consumed in the novel care pathway. The economic evaluation will also seek insights from qualitative research to better understand opportunity cost related to alternative care pathway. Finally, to help develop an evidence-informed care novel care pathway that is likely to be cost effective, the economic evaluation will bring together insights to identify principal components that will likely contribute the greatest amount towards intervention effectiveness. Working with the economist, resources related to ‘effective components’ will be considered and recommended for future intervention refinements.

Beyond assisting with development of this complex intervention, the economic evaluation of the ED PLUS intervention will be developed to include cost effectiveness analysis. The evaluation will aim to be conducted in a manner consistent with the guidelines published by the *Health and Information Quality Authority in Ireland* [68]. The objectives of the evaluation will be to identify, quantify and compare the cost and effect of the ED PLUS intervention relative to those under the status quo provision of care (i.e. usual care) and CGA alone (i.e. recommended best practice).

The incremental costs and effects of the programme relative to usual care will be calculated through incremental cost effectiveness analysis and will aim to calculating incremental cost-effectiveness ratio (ICER) [59]. The ICER is formally denoted by the following:

$$\mathrm{ICER}=\frac{{\mathrm{COST}}_{\mathrm{EDPlus}}-{\mathrm{COST}}_{\mathrm{UsualCare}}}{{\mathrm{EFFECT}}_{\mathrm{EDPlus}}-{\mathrm{EFFECT}}_{\mathrm{UsualCare}}}$$

For a robust estimate of cost effectiveness, COST and EFFECTS require mean averages estimated from definitive intervention study (which will be beyond the scope of this stage of research). Furthermore, it should be noted that COST should reflect the total net cost related to care and therefore is the sum of cost of intervention (i.e. providing ED PLUS, Usual Care or CGA) plus the cost consequences elsewhere (i.e. increases or decreases in service-use related to the treatment decision).

Collection of salient cost consequences will be piloted to inform the feasibility study by collecting data in each group at each time point using the Client Service Receipt Inventory (CSRI) questionnaire [69]. Cost consequences require a sample size calculation for cost effectiveness analysis [19, 70] and, whilst this pilot may provide some insights to an initial calculation, it will be recommended that a full power calculation be an intended outcome of the feasibility RCT.

### Qualitative evaluation

The qualitative evaluation will assess the acceptability of the trial methods, evaluate the acceptability of the ED PLUS, identify modifications to ED PLUS and describe participants experiences of ED PLUS [71]. One-to-one semi-structured telephone interviews with the dedicated research nurse, with trial participants in intervention arm 2, all medical and HSCPs involved in the intervention arm 2 will be undertaken on completion of the programme by a person independent of the trial. Participants will be contacted to undertake a telephone interview within one week of the completion of the ED PLUS intervention. All interviews will be digitally recorded and transcribed verbatim. All participants will be offered an opportunity to view their own transcript. A sample of the interview schedule is included in Appendix 2.

#### Data analysis

All data will be analysed and reported in accordance with the Consolidated Standards of Reporting Trials (CONSORT) guidelines for pilot and feasibility trials [72] and the CONSORT extension for reporting of patient-reported outcomes [73]. As this is a feasibility study with a relatively small sample size, formal hypothesis testing is not appropriate; rather, the purpose of any analyses will be to generate estimates to inform the planning of the definitive future trial. The analysis will be completed in two stages. Stage one will summarise the feasibility outcomes. Stage two will summarise the clinical outcomes data at 6 weeks and 6 months. As it is inappropriate to use feasibility trial data to formally test for between-group treatment effects, the analyses will primarily be of a descriptive nature [42, 72]. Descriptive statistics of the clinical outcomes data will be produced for each trial arm. Interval estimates of the potential intervention effects, relative to CGA and usual care only, will be produced in the form of a 95% confidence interval, to ensure that the effect size subsequently chosen for powering the definitive trial is plausible. These will refine the design characteristics of the future definitive trial.

#### Qualitative analysis

Interview data will be transcribed in full and analysed using a reflexive approach to thematic analysis [74] which will acknowledge and consider the centrality of researcher subjectivity. Reflexivity will enable the researcher team to consider and analyse how subjective and intersubjective elements influence the research process. Analysis will be facilitated through the use of NVIVO 12 software. When coding is complete, both participants and HSCPs will be invited to review and discuss preliminary analysis of the interview data and contribute to the process of identifying themes. Participants will also be invited to co-write/design a lay summary and infographic of the findings. Group video/phone conferences or one-to-one telephone calls will be scheduled to enable participants to contribute to analysis.

#### Fidelity

We will ensure fidelity to the research protocol through several means. First, the study coordinator will review baseline enrolment data on a weekly basis. Second, the study team will review the  proportion of subjects who are eligible, approached, consent to participate and enrolled on a weekly basis. Reasons for refusal are tracked to allow the study team to modify how they communicate study details when they approach the participant and for subsequent study planning. The primary investigator (PI) will hold weekly meetings with the research staff to discuss study progress and address concerns.

### Determining progression to the full trial

We shall progress to a full trial application if minimum success criteria are achieved in key feasibility aims and objectives. These criteria:

- A minimum of 80% recruitment of eligible patients
- A minimum of 80% completion rate of key outcome measures (including follow-up).

### Trial oversight and monitoring

Given the nature of this trial, the Trial Steering Committee (TSC) will comprise of the project manager (MC), PI (RG), key co-applicants, ED PLUS and other key external members of staff involved in the study. Specifically, the TSC will be responsible for (i) protocol development, (ii) obtaining ethical approval (iii) clinical set-up, on-going management, promotion of the study and for the interpretation and publishing of the results. The TSC will provide overall supervision of the study, in particular by monitoring study progress, and provide public, clinical, and professional advice, with pre-agreed terms of reference.

### Safety

In the light of COVID-19 pandemic, additional measures to increase safety of all treating staff and participants will be ensured. All patients are screened for COVID-19 on presentation to the ED and are all tested. All participants will be screened via telephone the day before a home visit to confirm the participant and all people living with them are free from signs and symptoms of COVID-19. All research staff will follow the COVID-19 protocols within UHL and the National Protocol for workers. All clinical and research staff will be fully equipped with Personal Protective Equipment (PPE) and are accustomed to use of PPE since the commencement of the pandemic in March 2020.

### Data management, audit and monitoring

All relevant data entered by a research nurse will be stored on Excel and pseudo-anonymised. The key to this pseudo-anonymisation will be kept by the PI [37]. A quality check of 20% of data will be completed by an independent researcher. If there is more than 5% of errors identified across the data entry, all data will be independently checked by the second independent researcher. For the life of the study, the pseudo-anonymised data will be stored on an encrypted and password protected electronic data capture system (CASTOR). Each member of the research team that is designated the task of entering data will have their own unique login and password for this system. Hard copy study-related materials including patient data will be stored in a secure locked environment with restricted access.

The confidentiality of the data will be always ensured by the PI and all members of the research team. Identifiable data will not be disclosed to third parties, and no participant’s name will appear in any of the results, as indicated in the participant’s information leaflet. Each participant in the study will be assigned a numerical code to link data collected at baseline to the data collected at follow-up at 6 weeks and 6 months. Aggregate data will be pseudo-anonymised. The research team will ensure anti-virus software is installed and up to date on all devices involved in data entry. All information trafficked from the clinical site to the research database will be pseudo-anonymised with unique study-specific subject numbers. Access to the research database is managed by the PI. No personal details or identifying data will be transferred from the hospital site to external sites, where the data will be analysed. Only coded/pseudo-anonymised data will be transferred to external sites.

### Dissemination plan

The results of this feasibility trial will inform the design of the anticipated definitive trial, rather than directly inform clinical decision-making, since clinical and cost effectiveness cannot be determined at this level. On completion, the results will be submitted for publication in open access peer-reviewed journals. Our PPI panel of older adults will assist in the dissemination in non-academic frameworks. All participants will be offered a lay summary of the results and a clinical summary will be presented to the ED clinical teams. A major output will be an application for funding for a future definite RCT, should the criteria for progression be achieved.

## Discussion

Rising ED visits and an ageing population with chronic health issues render ED interventions to reduce adverse outcomes in older adults a research priority. An ED visit is a key event which signals a deteriorating functional status [75]. Early identification in the ED [19] of older adults at risk of adverse outcomes is a key research priority. CGA is considered the gold standard for the assessment of older adults, but there is insufficient evidence to support its implementation on the ED at present. A key research priority defined by Kings Fund and Veteran Affairs Reports is to develop user informed transitional interventions to bridge the ED and community [16]. The ED PLUS intervention has been developed with the aim of addressing this important issue. One of the strengths of ED PLUS is that is has been developed with clinical practitioners, researchers and a PPI panel of older adults. Best practice guidelines recommend the need to test the feasibility and acceptability of RCT procedures prior to undertaking a definite trial. Guidelines for the management on patients during COVID 19 will be adhered to. This pilot feasibility RCT will provide important operational data into the practicality of undertaking a definite RCT including estimates of recruitment, attrition, baseline assessment scores and completion rates of the measures.

### Trial status

This is version 1 of the protocol (16^th^ September 2021). Recruitment is open. Any deviations to this protocol will be submitted to the respective ethics boards and updated on Clinical Trials Protocols and Results System (clinicaltrials.gov), and the changes will be discussed on dissemination of the results.

## Supplementary Information

**Additional file 1: Appendix 1.** ED PLUS demographic questionnaire

**Additional file 2: Appendix 2.** Interview guide ED PLUS participants

## Data Availability

Non-applicable.

## Abbreviations

AMAU: Acute medical assessment unit
CGA: Comprehensive geriatric assessment
CSRI: Client Service Receipt Inventory
ED: Emergency department
GP: General practitioner
HSE: Health Service Executive
ICER: Incremental cost-effectiveness ratio
ISAR: Identification of seniors at risk
MRC: Medical Research Council
PI: Primary Investigator
PPE: Personal protective equipment
PPI: Public and patient involvement
RCT: Randomised controlled trial
UHL: University Hospital Limerick
