# Supplementary material for: A physiotherapy-led transition to home intervention for older adults following emergency department discharge: protocol for a pilot feasibility randomised controlled trial
Source: Pilot Feasibility Stud. 2022 Jan 3;8:3. doi: 10.1186/s40814-021-00954-5 (PMC8720939; doi:10.1186/s40814-021-00954-5)
Supplement: Supplementary file 1 — Additional file 1: Appendix 1. ED PLUS demographic questionnaire [file 40814_2021_954_MOESM1_ESM.docx]

**ED PLUS**

**DEMOGRAPHIC QUESTIONNAIRE**

**Are you…?** Male/Female **What is your age?** ………….

**What is your ethnicity?** White Irish **What is your marital status?**

White Irish Traveller Married

Other white background In a relationship (not married)

Black Irish Single

Other black background Widowed

Asian Separated/Divorced

Any other mixed background Other_____________

**What is your highest level** Primary school **Residential status**

**Of educational attainment?** Secondary school lives alone

Third level/higher education lives with family/lives with who?

Post grad cert/diploma NH resident

Post grad masters/doctorate Other

**Have you fallen in the past** YES/NO **Do you smoke? YES/NO?**

**3 months?** If yes, for how long_________

**Do you drink Alcohol**? YES/NO **Source of referral**__________

Units per week ________________

**Presenting problem** ________________ **Mode of arrival to ED_____**

**Medications ________________ ________________________**

(type, dose, frequency) ________________ ________________________

________________ ________________________

________________ ________________________

**Referrals for services in ED** ________________ **Covid test results _________**
