# Supplementary material for: A physiotherapy-led transition to home intervention for older adults following emergency department discharge: protocol for a pilot feasibility randomised controlled trial
Source: Pilot Feasibility Stud. 2022 Jan 3;8:3. doi: 10.1186/s40814-021-00954-5 (PMC8720939; doi:10.1186/s40814-021-00954-5)
Supplement: Supplementary file 2 — Additional file 2: Appendix 2. Interview guide ED PLUS participants [file 40814_2021_954_MOESM2_ESM.zip › APPENDIX 2 Interview Guide ED PLUS participantsR1.docx]

**Interview Guide ED PLUS Participants**

Hi, (Participant Name) thank you for talking to me about your experience in the ED and your follow up care in your home/ So (Participant Name), we are going to talk to you about your experience in the ED.

- I wonder if you would tell me about what brought you to the ED on that day.

**Prompt:** Reason for attending GP Referral, Reassurance, Pain, no GP available to see you

- Did covid affect your attendance/did it delay your attendance to the ED?

**Prompt:** Can you tell a little bit about the concerns you would have had.

- Can you tell me about who you would have seen in the ED?

**Prompts:** Physiotherapist, Occupational Therapist, Nurse, Doctor,

- Can you tell me about what they would have talked to you about? What would they have done for/with you while you were there?
- Can you tell me about your experience of communicating with the healthcare staff in the ED?

**Prompts:** Do you feel you knew enough about the care you received? Is there any other information you would like to have gotten while there or afterwards? Who would you like to receive this information form?

- Did covid affect your ability to communicate with healthcare staff?

**Prompt:** Can you tell me a little bit about why that was? Prompt: PPE, Less time.

- Can you tell me about the care you received to prepare you for discharge from the ED?
- **Prompts:** Information on Prescription, Plans for follow-up care, Point of contact if needed, Primary Care Services
- Did you meet Mairéad Conneely, Physiotherapist, who called to your home, in the ED
- Can you tell me about the visit from Mairéad Conneely, and the care you received following your ED visit? / How did you feel about the ED PLUS intervention, where Mairéad Conneely called to your home to see you?

**Prompts:** Calling to you home, were you comfortable with the call to your home, did she arrange any other OPD visits?

- Were you happy that Mairéad was a physiotherapist?
- Did covid affect your follow-up care in any way?

**Prompt:** Can you tell me a little more about that? Mairéad wore PPE

- How did you feel about the treatment you received in your home?
- Can you tell me what Mairéad did in your home?

**Prompt:** did she look at you walking, look at your bed/bathroom, watch you do activities

- Who else did you talk to as part of Mairéad’s visit?

**Prompt:** OT, Dietician, Geriatrician

- Did your experience at this ED visit differ from previous ED visits? Can you tell me a little about why it was different?
- What would you tell your family about Mairéad’s visit?
- Overall, could you rate your experience from 0-10?
- Is there any way that the care you received could have been improved?

**Prompt:** Timely Care, More information.

**Further Prompts**

Can you tell me a little more about that experience?

Can you describe that further?

Would that have been similar to other experiences you would have had in the ED?
