# Supplementary material for: A physiotherapy-led transition to home intervention for older adults following emergency department discharge: protocol for a pilot feasibility randomised controlled trial
Source: Pilot Feasibility Stud. 2022 Jan 3;8:3. doi: 10.1186/s40814-021-00954-5 (PMC8720939; doi:10.1186/s40814-021-00954-5)
Supplement: Supplementary file 2 — Additional file 2: Appendix 2. Interview guide ED PLUS participants [file 40814_2021_954_MOESM2_ESM.zip › APPENDIX 2 Interview Guide ED PLUS staffR1.docx]

**Interview Guide ED PLUS**

To start, can you describe your role within the ED PLUS team?

From your perspective, did you find any aspect of your involvement challenging? Can you describe why? **Prompts:** Social Influences: Relationships between teams, Relationships between MDT team members.

How did the environment and challenging circumstances (Overcrowding, lack of community services, stress levels) impact on your role within the team or your practice? Can you describe how you overcame these challenges? **Prompts:** Referrals, Team communication.

Did anything in particular facilitate your involvement/ role within the team? Can you describe how this assisted you? **Prompts:** Communication, Regular Meetings, Clear Protocol.

Did covid impact on the way in which you interacted with the participants? **Prompt:** Time, Infection Control, Restricted from Recruiting Potential Participants, Follow-up.

How did you and the wider ED PLUS team compensate for this impact?

From your perspective, how did the ED PLUS team interventions impact on the care delivered to participants? Did any particular patient cohort benefit from the process? (Timely Intervention, Follow-up, Early Specialist Review)

From your perspective, how can we attain buy in from staff to get involved/ sustain involvement should a similar intervention be employed in the future? **Prompt:** Sustain Motivation.

Do you feel there was knowledge/understanding around your role/the role of the SOLAR team? **Prompt:** Team motivations, Team goals. If not, how can this understanding be enhanced.

If this team based intervention was employed in the future, would you make any adaptations, changes to your role or the teams approach?

Should all participants over the age of 65 be suitable to be seen (Including surgical, orthopaedic etc) would this be challenging and why?

Do you feel that the ED PLUS trial impacted on other care delivery or the allocation of patients? If yes can you describe how and why?

Did the diversity of the patient cohort impact on the teams role with a patient? For example, minimal time intervention with some participants, more with others. Can you describe why? i.e Frail well vs frail unwell.

Is there anything you would like to add?
